# Risk factors for hypoglycemia in patients with type 2 diabetes mellitus: a systematic review and meta-analysis

**DOI:** 10.3389/fendo.2026.1896142

**Published:** 2026-08-05

**Authors:** Meijin Li, Jiayao Liu, Pei Sun, Xinan Zhao, Chenyao Fan, Xiaoxia Guo, Wenting Zhao, Xiaoyan Guo

**Affiliations:** 1 School of Nursing, Changzhi Medical College, Changzhi, Shanxi, China; 2 Department of Neurosurgery, the Affiliated Heping Hospital of Changzhi Medical College, Changzhi, Shanxi, China

**Keywords:** hypoglycemia, meta-analysis, risk factors, systematic review, type 2 diabetes

## Abstract

**Introduction:**

This systematic review and meta-analysis aimed to identify risk factors for hypoglycemia in adults with type 2 diabetes.

**Methods:**

We searched PubMed, Embase, Cochrane Library, Web of Science, CNKI, Wanfang, VIP, and SinoMed from inception to July 8, 2026. Observational studies reporting risk factors for hypoglycemia were included. Study quality was assessed using the Newcastle-Ottawa Scale or AHRQ checklist. Odds ratios (ORs) were pooled using random- or fixed-effects models.

**Results:**

Ten studies (n = 4,945; hypoglycemia 1,647; non- hypoglycemia 3,298) were included. Longer diabetes duration (OR 2.67, 95% CI 1.81–3.95), insulin therapy (OR 2.46, 95% CI 1.93–3.15), comorbidities (OR 1.79, 95% CI 1.17–2.75), and polypharmacy (OR 4.73, 95% CI 3.07–7.30) were associated with higher odds of hypoglycemia. Higher HbA1c (OR 0.59, 95% CI 0.44–0.81) and self- monitoring of blood glucose (OR 0.52, 95% CI 0.49–0.56) were associated with lower odds. Body mass index showed a non- significant inverse association (OR 0.69, 95% CI 0.47–1.03). Heterogeneity varied across factors, and small- study effects were suggested for comorbidities.

**Discussion:**

These findings highlight interacting disease- , treatment- , and management- related determinants of hypoglycemia risk and may inform individualized risk assessment, although prospective studies are needed to validate these observations.

**Systematic Review Registration:**

https://www.crd.york.ac.uk/prospero/, identifier CRD420251146701.

## 1 Introduction

Diabetes mellitus is among the most pressing global public health challenges of the 21st century, imposing substantial burdens through premature mortality, disability, and rising healthcare costs (1, 2). The global prevalence of diabetes continues to increase, and contemporary projections suggest that the number of affected adults will exceed 1.3 billion by 2050 (3). Type 2 diabetes mellitus (T2DM) accounts for the vast majority of cases worldwide and remains the principal driver of diabetes-related morbidity (3).

Glycemic control is central to T2DM management (4). However, intensification of glucose-lowering therapy can lead to iatrogenic hypoglycemia, particularly with insulin and insulin secretagogues (5). Hypoglycemia is increasingly recognized not merely as an adverse effect but as a clinically consequential event linked to poorer outcomes (6, 7). Observational evidence indicates that hypoglycemia is associated with higher risks of cardiovascular events and death in individuals with diabetes (8), and severe episodes impose additional psychosocial harms, including fear of hypoglycemia and impaired quality of life (9). In parallel, hypoglycemia contributes to avoidable healthcare utilization. Although administrative data capture only a fraction of severe events, population-based studies consistently show that hypoglycemia-related emergency department visits and hospitalizations occur at clinically meaningful rates, with higher incidence among insulin- and sulfonylurea-treated patients (10). As a result, clinicians and guideline developers lack a consolidated estimate of which factors most robustly predict hypoglycemia across settings.

Over the past decade, numerous observational and interventional studies have examined risk factors for hypoglycemia in T2DM, including demographic characteristics, treatment regimens, and comorbid conditions (11–14). However, the reported associations have been heterogeneous and sometimes conflicting across studies. For key clinical variables—such as overall glycemic control, age, renal function, and specific drug classes—some reports suggest a strong relationship with hypoglycemia risk, whereas others find only modest or no associations (15–17). These inconsistencies may reflect differences in study populations, definitions and ascertainment of hypoglycemia, and study design.

To address these gaps, we performed a systematic review and meta-analysis to synthesize and quantify the evidence on risk factors for hypoglycemia among patients with T2DM. By clarifying the strength and consistency of associations across studies, our findings aim to support risk stratification in clinical practice and facilitate targeted prevention strategies to reduce hypoglycemic events and their downstream complications.

## 2 Methods

### 2.1 Protocol and registration

The protocol for this review was preregistered on PROSPERO (ID: CRD420251146701), and the findings are reported in accordance with Preferred Reporting Items for Systematic Reviews and Meta-Analyses (PRISMA) guidelines (18).

### 2.2 Selection criteria and search strategy

The inclusion criteria were as follows: (1) Study design: observational studies, including cohort, case–control, or cross-sectional studies; (2) Population: adults with a diagnosis of type 2 diabetes; (3) Study focus: evaluation of risk factors or predictors of hypoglycemic events in patients with type 2 diabetes; (4) Data reporting: studies that reported odds ratios (ORs) with corresponding 95% confidence intervals (95%CI) or provided sufficient raw data to calculate these estimates.

Studies were excluded if they met any of the following criteria: (1) abstract-only publications or studies for which the full text was unavailable; (2) publications in languages other than Chinese or English; (3) insufficient data to extract or calculate effect estimates required for meta-analysis; (4) conference abstracts, narrative reviews, systematic reviews, guidelines, editorials, letters, or case reports; (5) studies rated as assessed by the NOS or AHRQ criteria (see Quality assessment section).

Databases were searched (PubMed, Embase via Ovid, the Cochrane Library, Web of Science, China National Knowledge Infrastructure (CNKI), Wanfang, VIP Chinese Science and Technology Journal Database (VIP), and SinoMed) using combinations of subject headings, keywords, and MeSH terms related to ‘type 2 diabetes’, ‘hypoglycemia’, and ‘risk factors’ (the complete search strategies for each database are provided in online **Supplementary Table 1**). The search was restricted to peer-reviewed journal articles published in English or Chinese from database inception to July 8, 2026. English-language databases were searched using English terms, while Chinese-language databases were searched using corresponding Chinese terms. In addition, we searched the PROSPERO international prospective register of systematic reviews to identify similar ongoing or completed reviews and to minimize duplication of work. We did not search grey literature, conference proceedings, dissertation databases, or trial registries. However, we manually screened the reference lists of all included studies and relevant prior systematic reviews to identify additional eligible studies not captured by the database searches. Any potentially relevant records identified through this hand-searching process were assessed against the same inclusion criteria.

### 2.3 Data extraction

All search results were imported into EndNote X9 (Clarivate, Philadelphia, Pennsylvania, USA) for duplication. Two reviewers independently screened titles/abstracts and full texts, extracted data and assessed risk of bias. All screening and data extraction were conducted in duplicate, with any disagreements resolved through discussion and, when necessary, consultation with a third reviewer (the corresponding author).

A standardized data extraction form was used to collect information on study characteristics (study title, first author, years of publication, country, and study design), sample size, risk factors, and reported effect estimates (odds ratios [ORs], and 95% confidence intervals [CIs]).Across the included studies, all uniformly employed <3.9 mmol/L as the glycemic threshold for defining non-severe hypoglycemia and focused on comparisons with no hypoglycemia. When relevant data were missing or unclear, we attempted to contact the corresponding authors of the original studies for clarification or additional information.

### 2.4 Quality assessment (risk of bias)

Methodological quality of cohort and case–control studies was assessed using the Newcastle–Ottawa Scale (NOS) (19), which evaluates three domains: selection of study groups, comparability of groups, and ascertainment of exposure or outcome. Studies were classified as high (7–9 points), moderate (4–6 points), or low (0–3 points) quality based on the total NOS score. For cross-sectional studies, we used the 11-item checklist developed by the Agency for Healthcare Research and Quality (AHRQ) (20). Each item was scored as ““yes” (1 point), “no,” or “unclear,” and overall quality was categorized as high (8–11 points), moderate (4–7 points), or low (0–3 points). Quality assessments were conducted independently by two reviewers, and any discrepancies were resolved by discussion or, if necessary, consultation with a third reviewer.

### 2.5 Statistical analysis

Meta-analyses were performed for risk factors reported in at least three independent studies. Pooled effect estimates were calculated as odds ratios (ORs) with corresponding 95% confidence intervals (CIs). All included studies reported adjusted odds ratios derived from multivariate regression models, and these adjusted estimates were extracted for the primary meta-analysis. When ORs and 95% CIs were not directly reported, they were derived from the available raw data using standard methods; no statistical imputation was undertaken for missing data. All primary meta-analyses were conducted using Review Manager (RevMan) version 5.4 (The Cochrane Collaboration), and tests for publication bias were performed in Stata version 17.0 (StataCorp, College Station, TX, USA). Odds ratios (ORs) with corresponding 95% confidence intervals (CIs) were used as the summary effect measure. When ORs were not explicitly provided, they were calculated from available raw data using standard methods; no imputation was performed for missing values.

Statistical heterogeneity across studies was assessed using Cochran’s Q test and quantified with the I² statistic. A fixed-effects model was used when heterogeneity was low (I² < 50% and P ≥ 0.10 for Q), whereas a random-effects model (DerSimonian–Laird method) was applied when substantial heterogeneity was present (I² ≥ 50% or P < 0.10). To evaluate the robustness of the pooled estimates, sensitivity analyses were conducted by alternating between fixed- and random-effects models and by sequentially omitting one study at a time (leave-one-out analysis).

Potential publication bias was evaluated using Egger’s regression test, which was performed only when ten or more studies were available for a given risk factor. A two-sided P value > 0.05 was interpreted as indicating no statistically significant small-study effect.

Owing to the limited number of studies within most risk factor categories, subgroup analyses and meta-regression were not undertaken. The study selection process was documented using a PRISMA flow diagram, and details of study characteristics, quality assessments, and quantitative synthesis results are summarized in structured tables.

## 3 Results

### 3.1 Literature search results

**Figure 1:**
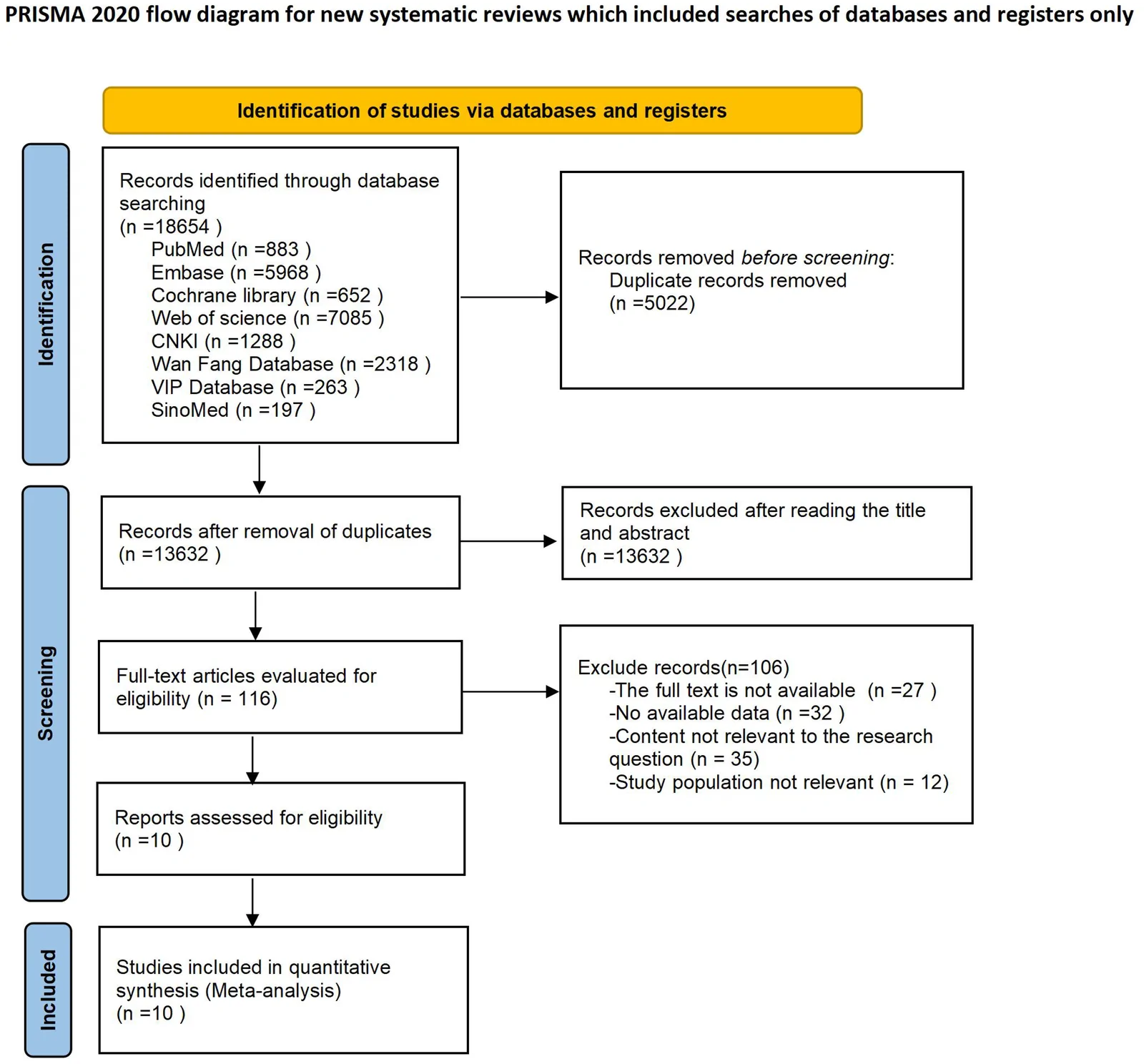
PRISMA flow diagram.

**Figure 1** illustrates the PRISMA flow chart. A total of 18654 records were identified and 5022 duplicates were removed. The titles and abstracts of 13632 records were screened, of which 13632 were excluded. Full texts of 116 reports were assessed for eligibility, and 106 were excluded for the following reasons: full text not available (n = 27), insufficient data for extraction (n = 32), not relevant to the research question (n=35), and ineligible study population (n=12). Ultimately, 10 studies were included in the systematic review and meta analysis.

### 3.2 Characteristics of included studies

Ten studies were included, comprising a total of 4,945 patients with type 2 diabetes. According to the original reports, 1,647 patients experienced hypoglycemia and 3,298 did not. The studies were conducted in China, Indonesia, Spain, Saudi Arabia, the United States, and Jordan, and included five cohort studies, three case–control studies, and two cross-sectional studies. Sample sizes of individual studies ranged from 267 to 862 participants, and all studies compared patients with and without hypoglycemia. The main risk factors investigated included duration of diabetes, HbA1c, insulin therapy, comorbidities/diabetic complications, BMI, self-monitoring of blood glucose (SMBG), polypharmacy, regular exercise/physical activity, oral hypoglycemic agents, history of hypoglycemia, age, sex, cognitive impairment, and family history of diabetes. The detailed characteristics of each study are summarized in **Table 1**.

**Table 1:** Characteristics of included studies and investigated risk factors.

Study	Country	Study design	Sample size		Risk factors
			H	NH	
Hou 2024 (20)	China	case-control study	193	392	②④⑦⑪⑬
Zuo 2021 (21)	China	case-control study	205	561	①②⑤⑧
Veryanti 2025 (22)	Indonesia	cross-sectional	267	234	②④⑨
Kong 2019 (23)	China	cross-sectional	180	189	①③⑤⑥⑨⑩
Tomás 2023 (24)	Spain	cohort study	154	170	②③⑤⑫
Alghamdi 2020 (25)	Saudi Arabia	case-control study	152	174	③⑧
Zhang 2020 (26)	China	cohort study	67	221	①③⑥⑦
Zhang 2024 (27)	China	cohort study	225	321	①③④⑥⑩⑭
Timple 2025 (28)	the United States	cohort study	65	535	④⑦⑪
Ahmad 2024 (29)	Jordan	cohort study	139	501	③④⑮

① Course of disease; ② HbA1c; ③ Insulin therapy; ④ Comorbidities; ⑤ BMI; ⑥ Self-monitoring of blood glucose; ⑦ Polypharmacy; ⑧ Diabetic complications; ⑨ Regular exercise; ⑩ Oral hypoglycemic agents; ⑪ History of hypoglycemia; ⑫ Age; ⑬ Gender; ⑭ Cognitive impairment; ⑮ Family history of diabetes.

### 3.3 Study quality assessment

The ten included studies comprised five cohort studies, three case-control studies (all assessed with the Newcastle-Ottawa Scale), and two cross-sectional studies (assessed with the AHRQ checklist). NOS total scores for cohort and case–control studies ranged from 6 to 8, indicating moderate to high methodological quality. The two cross-sectional studies achieved AHRQ scores of 7 and 8, also reflecting moderate to high quality. Detailed quality assessment results are provided in **Tables 2** and **3**.

**Table 2:** Risk of bias assessment of cohort and case–control studies using the Newcastle–Ottawa Scale (NOS).

Study	Selection (0-4)	Comparability (0-2)	Exposure/ Outcome (0-3)	Total scores (0-9)
Hou 2024 (20)	4	1	3	8
Zuo 2021 (21)	4	1	2	7
Tomás 2023 (24)	4	2	2	8
Alghamdi 2020 (25)	3	1	2	6
Zhang 2020 (26)	3	1	2	6
Zhang 2024 (27)	4	1	2	7
Timple 2025 (28)	3	1	3	7
Ahmad 2024 (29)	4	1	3	8

**Table 3:** Risk of bias assessment of cross-sectional studies using the AHRQ checklist.

Study	Item 1-11											Total scores(0-11)
	①	②	③	④	⑤	⑥	⑦	⑧	⑨	⑩	⑪	
Veryanti 2025 (22)	Yes	Yes	Yes	Yes	No	Yes	Yes	Yes	No	Unclear	Unclear	7
Kong 2019 (23)	Yes	Yes	Yes	Yes	No	Yes	Yes	Yes	No	Yes	No	8

AHRQ, Agency for Healthcare Research and Quality. Cross-sectional study quality was assessed using the 11-item AHRQ checklist. Each item was scored as Yes = 1, No/Unclear = 0; total score ranges from 0 to 11, with higher scores indicating better methodological quality. ① Define the source of information (survey, record review); ② List inclusion and exclusion criteria for exposed and unexposed subjects (cases and controls) or refer to previous publications; ③ Indicate time period used for identifying patients; ④ Indicate whether or not subjects were consecutive if not population - based; ⑤ Indicate if evaluators of subjective components of study were masked to other aspects of the status of the participants; ⑥ Describe any assessments undertaken for quality assurance purposes (e.g., test/retest of primary outcome measurements); ⑦ Explain any patient exclusions from analysis; ⑧ Describe how confounding was assessed and/or controlled; ⑨ If applicable, explain how missing data were handled in the analysis; ⑩ Summarize patient response rates and completeness of data collection; ⑪ Clarify what follow - up, if any, was expected and the percentage of patients for which incomplete data or follow - up was obtained.

### 3.4 Results of the meta-analysis

Because the risk factors investigated varied across studies, quantitative synthesis was feasible for seven factors that were each reported in at least three studies: duration of diabetes, HbA1c level, insulin therapy, comorbidities, self-monitoring of blood glucose (SMBG), polypharmacy, and body mass index (BMI). Pooled estimates indicated higher odds of hypoglycemia with longer diabetes duration (OR 2.67, 95% CI 1.81–3.95; random-effects; I² = 61%), insulin therapy (OR 2.46, 95% CI 1.93–3.15; fixed-effects; I² = 12%), comorbidities (OR 1.79, 95% CI 1.17–2.75; random-effects; I² = 84%), and polypharmacy (OR 4.73, 95% CI 3.07–7.30; fixed-effects; I² ≈ 0%). Lower odds were observed for higher HbA1c (OR 0.59, 95% CI 0.44–0.81; random-effects; I² = 86%) and for SMBG (OR 0.52, 95% CI 0.49–0.56; fixed-effects; I² ≈ 0%). BMI showed an inverse but non-significant association (OR 0.69, 95% CI 0.47–1.03; random-effects; I² = 74%). Full summary ORs and 95% CIs are presented in **Table 4**.

**Table 4:** Meta-analysis of risk factors for hypoglycemia: pooled odds ratios, heterogeneity, and publication bias.

Risk factor	Studies (n)	Heterogeneity		Effect model	Meta - analysis results		Egger’s P value
		*I^2^* (%)	*P* value		*OR*(95%CI)	*P* value	
Duration of diabetes	4	61	0.05	Random	2.67 [1.81,3.95]	<0.001	0.467
HbA1c	4	86	<0.001	Random	0.59 [0.44,0.81]	0.001	0.088
Insulin therapy	6	12	0.34	Fixed	2.46 [1.93,3.15]	<0.001	0.076
Diabetic complications	5	84	<0.001	Random	1.79 [1.17,2.75]	0.008	0.016
BMI	3	74	0.02	Random	0.69 [0.47,1.03	0.07	0.217
SMBG	3	0	1	Fixed	0.52 [0.49,0.56]	<0.001	0.491
Polypharmacy	3	0	0.86	Fixed	4.73 [3.07,7.30]	<0.001	0.723

SMBG, Self-monitoring of blood glucose. OR > 1 indicates higher odds of hypoglycemia; *OR* < 1 indicates lower odds. Effect model was chosen according to prespecified criteria (random-effects when substantial heterogeneity was present; otherwise fixed-effects). Egger’s test *p* < 0.10 suggests potential small-study effects; given the limited number of studies per factor, results should be interpreted with caution.

### 3.5 Sensitivity analysis and publication bias

Sensitivity analyses showed that the pooled estimates for duration of diabetes, HbA1c, insulin therapy, diabetic complications, SMBG, and polypharmacy were generally robust to model choice (fixed vs. random effects) and to leave-one-out analyses. In contrast, the association between BMI and hypoglycemia was unstable and changed noticeably when different models were applied or when individual studies were omitted.

Publication bias was assessed using Egger’s test for risk factors with a sufficient number of studies. Evidence of small-study effects was observed for diabetic complications (P < 0.05), whereas no significant publication bias was detected for the other risk factors (P > 0.05). As prespecified, no subgroup analyses or meta-regression were performed owing to the limited number of studies per risk factor. These findings are summarized in **Table 4**.

### 3.6 Descriptive analysis of risk factors

A narrative analysis was undertaken for risk factors that were reported in fewer than three studies or for which meta-analysis was not feasible. Diabetic complications (21, 25) and a history of hypoglycemia were each associated with an increased risk of hypoglycemia in two independent studies (20, 28). Two studies suggested that higher levels of regular physical activity or exercise were related to a higher occurrence of hypoglycemia (22, 23). Additional potential risk factors reported in single studies included cognitive impairment, older age, and a family history of diabetes (24, 27, 29). Although these findings suggest that multiple clinical and behavioral factors may influence hypoglycemia risk in patients with type 2 diabetes, the evidence for these factors remains limited and requires confirmation in future studies.

## 4 Discussion

In this meta-analysis of 10 observational studies (n = 4,945), seven risk factors were quantitatively synthesized. Longer diabetes duration, insulin therapy, comorbidities, and polypharmacy were associated with higher odds of hypoglycemia, whereas higher HbA1c and self-monitoring of blood glucose (SMBG) were associated with lower odds. For BMI, the pooled OR was 0.69, but its 95% confidence interval crossed 1.0, with considerable heterogeneity (I² = 74%) and unstable results in sensitivity analyses. Differences across studies in obesity phenotypes (central vs. general adiposity), concomitant medication dosages, and comorbidity profiles may have confounded the association. Further investigations are warranted to clarify the impact of adiposity patterns on hypoglycemia risk. Heterogeneity varied across factors, and small-study effects were detected for comorbidities. These findings underscore that hypoglycemia in type 2 diabetes arises from interacting disease-, treatment-, and management-related determinants and support individualized risk assessment and management.

### 4.1 Disease-related risk factors

Longer diabetes duration was associated with greater hypoglycemia risk (pooled OR = 2.67), consistent with prior evidence (30). Progressive β-cell dysfunction, accumulation of diabetes-related complications, and prolonged exposure to glucose-lowering therapies likely contribute to increased susceptibility (31). Patients with longer diabetes duration may benefit from closer surveillance and regimens that prioritize hypoglycemia minimization, although these suggestions require confirmation in future prospective studies. Lower HbA1c was inversely associated with hypoglycemia (pooled OR = 0.59), indicating fewer hypoglycemic events at higher HbA1c levels; conversely, stricter glycemic control increases hypoglycemia risk, aligning with previous studies (32, 33). Between-study heterogeneity was substantial (I² = 86%) but fell to 36% after excluding Zuo et al. (21), suggesting differences in design, HbA1c thresholds, or population characteristics. Given HbA1c reflects average glycemia over approximately three months, individualized targets should balance benefits and hypoglycemia risk. As suggested by Liu (34), maintaining HbA1c in a moderate range (e.g., 7.0% ~ 7.9%) may be associated with lower hypoglycemia risk in susceptible patients, particularly older adults, those with longer duration, or with comorbidities.

Comorbidities were associated with increased hypoglycemia risk (pooled OR = 1.79) with high heterogeneity (I²= 84%), which dropped to 19% after excluding Zhang et al. (27). This indicates that clinical and population differences can materially affect pooled estimates. Mechanistically, conditions such as renal impairment reduce insulin clearance and increase exposure, predisposing to severe hypoglycemia without dose adjustment (35); cognitive impairment hampers self-management and recognition of hypoglycemia, fostering recurrent episodes and further cognitive decline (36); and other comorbid conditions can alter insulin sensitivity or pharmacodynamics (37). Notably, Egger’s test suggested small-study effects for this factor, so the magnitude of association should be interpreted with caution. In practice, comorbidity-informed medication selection, dose adjustment, and tailored glycemic targets are warranted.

### 4.2 Treatment-related risk factors

Insulin therapy was associated with higher hypoglycemia risk (pooled OR = 2.46), consistent with the pharmacologic action of exogenous insulin and its sensitivity to mismatches with food intake, activity level, and interindividual variability in absorption and action (38, 39). Although regimen-specific comparisons were not feasible in our synthesis, prior work suggests higher hypoglycemia risk with premixed and prandial regimens compared with basal insulin (40). Where feasible, use of long-acting analogs, careful titration, and regimen simplification can mitigate risk (41).

Polypharmacy showed a strong association with hypoglycemia (pooled OR = 4.73). Potential mechanisms include pharmacokinetic or pharmacodynamic interactions that potentiate glucose-lowering effects or impair drug metabolism, leading to higher effective exposure (42, 43). Complex regimens also elevate the risk of nonadherence dosing errors, and unsupervised adjustments, destabilizing glycemic control (44). Regular medication reconciliation, avoidance of high-risk combinations, simplification of dosing schedules, and clear patient instructions are key mitigation strategies.

### 4.3 Management-related risk factors

SMBG was associated with lower odds of hypoglycemia (pooled OR = 0.52). Higher monitoring frequency has been linked to fewer hypoglycemic events (45), likely through two mechanisms: early detection of downward glucose trends enabling timely carbohydrate intake, and provision of objective data to guide individualized treatment adjustments (46).Clinicians should emphasize the purpose and timing of SMBG, teach recognition of early symptoms, and provide action plans for impending hypoglycemia. Structured SMBG programs can empower patients and improve safety.

### 4.4 Strengths and limitations

This study synthesized data across multiple regions and designs and applied standardized quality tools (NOS and AHRQ), with most studies judged moderate-to-high quality. Nonetheless, several limitations merit consideration. First, all included studies were observational; residual confounding and reverse causation cannot be excluded. Second, the number of studies per risk factor was limited (often n = 3–6), reducing the precision of pooled estimates and precluding formal subgroup analyses or meta-regression. Third, although all included studies uniformly employed <3.9 mmol/L for defining non-severe hypoglycemia and focused on comparisons with no hypoglycemia, discrepancies existed in geographical regions (with most studies conducted in Asian populations) and participant sources (inpatient vs. outpatient), which may limit generalizability to other populations and contribute to heterogeneity. Leave-one-out sensitivity analyses suggested that specific studies contributed substantially to the high I² values for HbA1c and comorbidities, yet these findings remain exploratory due to the limited number of studies. Fourth, the definition of polypharmacy varied across studies (e.g., medication count thresholds, inclusion of non-antidiabetic agents, and over-the-counter drugs); the pooled OR of 4.73 should therefore be viewed as a summary association rather than a precise dose-response estimate. Finally, Egger’s test suggested small-study effects for comorbidities, and publication bias cannot be ruled out.

### 4.5 Clinical implications and future research

Our findings support a risk-stratified approach to hypoglycemia prevention in T2DM. Patients with longer disease duration, on insulin therapy, and with multiple comorbidities and medications should receive individualized glycemic targets, regimen choices that prioritize hypoglycemia safety (e.g., basal analogs where appropriate), dose adjustments based on renal function and daily patterns, and structured SMBG. Medication review and deprescribing strategies may reduce risk in those with polypharmacy. Future studies should standardize definitions of hypoglycemia, specify and compare insulin regimens and co-medications, and evaluate the dose–response relationships of SMBG and HbA1c targets within key subgroups (e.g., older adults, impaired renal function). Prospective cohorts and pragmatic trials, ideally incorporating continuous glucose monitoring, are needed to clarify causality and optimize risk-mitigation strategies.

## 5 Conclusion

Hypoglycemia in T2DM reflects converging disease, treatment, and management-related factors. Longer duration, insulin use, comorbidities, and polypharmacy increase risk, whereas SMBG and higher HbA1c targets within individualized ranges appear protective. Integrating these determinants into routine care can help tailor safer glycemic management and reduce hypoglycemia burden.

## Data Availability

The original contributions presented in the study are included in the article/**Supplementary Material**. Further inquiries can be directed to the corresponding author.
