# Correction: Risk factors for hypoglycemia in patients with type 2 diabetes mellitus: a systematic review and meta-analysis

**DOI:** 10.3389/fendo.2026.1988243

**Published:** 2026-09-15

**Authors:** Meijin Li, Jiayao Liu, Pei Sun, Xinan Zhao, Chenyao Fan, Xiaoxia Guo, Wenting Zhao, Xiaoyan Guo

**Affiliations:** 1 School of Nursing, Changzhi Medical College, Changzhi, Shanxi, China; 2 Department of Neurosurgery, the Affiliated Hospital of Changzhi Medical College, Changzhi, Shanxi, China

**Keywords:** hypoglycemia, meta-analysis, risk factors, systematic review, type 2 diabetes

The funder 2025 University-level Graduate Practical Innovation Project of Changzhi Medical College (grant number CY2025SJ07) to Meijin Li was erroneously omitted.

The original version of this article has been updated.

