# Supplementary material for: Risk factors for hypoglycemia in patients with type 2 diabetes mellitus: a systematic review and meta-analysis
Source: Front Endocrinol (Lausanne). 2026 Aug 5;17:1896142. doi: 10.3389/fendo.2026.1896142 (PMC13485724; doi:10.3389/fendo.2026.1896142)
Supplement: Supplementary file 1 [file Table1.docx]

**Supplementary Table S1**

| **Databases** |  | **search strategies** | **number of documents** |
| --- | --- | --- | --- |
| Pubmed | #1 | ((((((((((((((((((((((((((((((TS=(Diabetes mellitus, type 2)) OR TS=(T2DM)) OR TS=(Diabetes Mellitus, Stable)) OR TS=(Stable Diabetes Mellitus)) OR TS=(Diabetes Mellitus, Noninsulin Dependent)) OR TS=(Diabetes Mellitus, Adult-Onset)) OR TS=(Adult-Onset Diabetes Mellitus)) OR TS=(Diabetes Mellitus, Adult Onset)) OR TS=(Diabetes Mellitus, Ketosis-Resistant)) OR TS=(Diabetes Mellitus, Ketosis Resistant)) OR TS=(Ketosis-Resistant Diabetes Mellitus)) OR TS=(Diabetes Mellitus, Non Insulin Dependent)) OR TS=(Diabetes Mellitus, Non-Insulin-Dependent)) OR TS=(Non-Insulin-Dependent Diabetes Mellitus)) OR TS=(Diabetes Mellitus, Type II)) OR TS=(Diabetes Mellitus, Maturity-Onset)) OR TS=(Diabetes Mellitus, Maturity Onset)) OR TS=(Maturity-Onset Diabetes Mellitus)) OR TS=(Maturity Onset Diabetes Mellitus)) OR TS=(Diabetes Mellitus, Slow-Onset)) OR TS=(Diabetes Mellitus, Slow Onset)) OR TS=(Slow-Onset Diabetes Mellitus)) OR TS=(Type 2 Diabetes Mellitus)) OR TS=(Noninsulin-Dependent Diabetes Mellitus)) OR TS=(Noninsulin Dependent Diabetes Mellitus)) OR TS=(Maturity-Onset Diabetes)) OR TS=(Diabetes, Maturity-Onset)) OR TS=(Maturity Onset Diabetes)) OR TS=(Type 2 Diabetes)) OR TS=(Diabetes, Type 2)) OR TS=(Diabetes Mellitus, Noninsulin-Dependent) | 264248 |
|  | #2 | ((((((((TS=(hypoglycemia)) OR TS=(Fasting Hypoglycemia)) OR TS=(Hypoglycemia, Fasting)) OR TS=(Postabsorptive Hypoglycemia)) OR TS=(Hypoglycemia, Postabsorptive)) OR TS=(Postprandial Hypoglycemia)) OR TS=(Hypoglycemia, Postprandial)) OR TS=(Reactive Hypoglycemia)) OR TS=(Hypoglycemia, Reactive) | 33601 |
|  | #3 | (((((((((((((((((((((TS=(Risk Factors)) OR TS=(Factor, Risk)) OR TS=(Risk Factor)) OR TS=(Population at Risk)) OR TS=(Populations at Risk)) OR TS=(Risk Scores)) OR TS=(Risk Score)) OR TS=(Score, Risk)) OR TS=(Risk Factor Scores)) OR TS=(Risk Factor Score)) OR TS=(Score, Risk Factor)) OR TS=(Health Correlates)) OR TS=(Correlates, Health)) OR TS=(Social Risk Factors)) OR TS=(Factor, Social Risk)) OR TS=(Factors, Social Risk)) OR TS=(Risk Factor, Social)) OR TS=(Risk Factors, Social)) OR TS=(Social Risk Factor)) OR TS=(influencing factor*)) OR TS=(related factor*)) OR TS=(associated factor*) | 1380884 |
|  | #4 | #1 AND #2 AND #3 | 756 |
| web of science | #1 | ((((((((((((((((((((((((((((((TS=(Diabetes mellitus, type 2)) OR TS=(T2DM)) OR TS=(Diabetes Mellitus, Stable)) OR TS=(Stable Diabetes Mellitus)) OR TS=(Diabetes Mellitus, Noninsulin Dependent)) OR TS=(Diabetes Mellitus, Adult-Onset)) OR TS=(Adult-Onset Diabetes Mellitus)) OR TS=(Diabetes Mellitus, Adult Onset)) OR TS=(Diabetes Mellitus, Ketosis-Resistant)) OR TS=(Diabetes Mellitus, Ketosis Resistant)) OR TS=(Ketosis-Resistant Diabetes Mellitus)) OR TS=(Diabetes Mellitus, Non Insulin Dependent)) OR TS=(Diabetes Mellitus, Non-Insulin-Dependent)) OR TS=(Non-Insulin-Dependent Diabetes Mellitus)) OR TS=(Diabetes Mellitus, Type II)) OR TS=(Diabetes Mellitus, Maturity-Onset)) OR TS=(Diabetes Mellitus, Maturity Onset)) OR TS=(Maturity-Onset Diabetes Mellitus)) OR TS=(Maturity Onset Diabetes Mellitus)) OR TS=(Diabetes Mellitus, Slow-Onset)) OR TS=(Diabetes Mellitus, Slow Onset)) OR TS=(Slow-Onset Diabetes Mellitus)) OR TS=(Type 2 Diabetes Mellitus)) OR TS=(Noninsulin-Dependent Diabetes Mellitus)) OR TS=(Noninsulin Dependent Diabetes Mellitus)) OR TS=(Maturity-Onset Diabetes)) OR TS=(Diabetes, Maturity-Onset)) OR TS=(Maturity Onset Diabetes)) OR TS=(Type 2 Diabetes)) OR TS=(Diabetes, Type 2)) OR TS=(Diabetes Mellitus, Noninsulin-Dependent) | 612466 |
|  | #2 | ((((((((TS=(hypoglycemia)) OR TS=(Fasting Hypoglycemia)) OR TS=(Hypoglycemia, Fasting)) OR TS=(Postabsorptive Hypoglycemia)) OR TS=(Hypoglycemia, Postabsorptive)) OR TS=(Postprandial Hypoglycemia)) OR TS=(Hypoglycemia, Postprandial)) OR TS=(Reactive Hypoglycemia)) OR TS=(Hypoglycemia, Reactive) | 96034 |
|  | #3 | (((((((((((((((((((((TS=(Risk Factors)) OR TS=(Factor, Risk)) OR TS=(Risk Factor)) OR TS=(Population at Risk)) OR TS=(Populations at Risk)) OR TS=(Risk Scores)) OR TS=(Risk Score)) OR TS=(Score, Risk)) OR TS=(Risk Factor Scores)) OR TS=(Risk Factor Score)) OR TS=(Score, Risk Factor)) OR TS=(Health Correlates)) OR TS=(Correlates, Health)) OR TS=(Social Risk Factors)) OR TS=(Factor, Social Risk)) OR TS=(Factors, Social Risk)) OR TS=(Risk Factor, Social)) OR TS=(Risk Factors, Social)) OR TS=(Social Risk Factor)) OR TS=(influencing factor*)) OR TS=(related factor*)) OR TS=(associated factor*) | 8767651 |
|  | #4 | #1 AND #2 AND #3 | 7085 |
